# Reduction in number to treat versus number needed to treat

**DOI:** 10.1186/s12874-021-01246-5

**Published:** 2021-03-09

**Authors:** Chenyang Zhang, Guosheng Yin

**Affiliations:** Department of Statistics and Actuarial Science, The University of Hong Kong, Pokfulam Road, Hong Kong, SAR China

**Keywords:** Absolute risk reduction, Number needed to treat, Randomized controlled trials, Restricted mean survival time, Treatment effect

## Abstract

**Background:**

We propose a new measure of treatment effect based on the expected reduction in the number of patients to treat (RNT) which is defined as the difference of the reciprocals of clinical measures of interest between two arms. Compared with the conventional number needed to treat (NNT), RNT shows superiority with both binary and time-to-event endpoints in randomized controlled trials (RCTs).

**Methods:**

Five real RCTs, two with binary endpoints and three with survival endpoints, are used to illustrate the concept of RNT and compare the performances between RNT and NNT. For survival endpoints, we propose two versions of RNT: one is based on the survival rate and the other is based on the restricted mean survival time (RMST). Hypothetical scenarios are also constructed to explore the advantages and disadvantages of RNT and NNT.

**Results:**

Because there is no baseline for computation of NNT, it fails to differentiate treatment effect in the absolute scale. In contrast, RNT conveys more information than NNT due to its reversed order of differencing and inverting. For survival endpoints, two versions of RNT calculated as the difference of the reciprocals of survival rates and RMSTs are complementary to each other. The RMST-based RNT can capture the entire follow-up profile and thus is clinically more intuitive and meaningful, as it inherits the time-to-event characteristics for survival endpoints instead of using truncated binary endpoints at a specific time point.

**Conclusions:**

The RNT can serve as an alternative measure for quantifying treatment effect in RCTs, which complements NNT to help patients and clinicians better understand the magnitude of treatment benefit.

**Supplementary Information:**

The online version contains supplementary material available at 10.1186/s12874-021-01246-5.

## Background

Randomized controlled trials (RCTs) are the gold standard to evaluate treatment effect of a new intervention in comparison with a control (e.g., the standard of care) [1]. However, it is often difficult to convey the findings in RCTs to patients and clinicians due to the complexity of statistical analysis and lack of interpretability of measurements of treatment effect. For example, let *p*_*E*_ and *p*_*C*_ denote the response rates of the experimental treatment and control, respectively. The relative risk RR = *p*_*E*_/*p*_*C*_ is the ratio of two response rates, absolute risk reduction ARR = *p*_*E*_ − *p*_*C*_ corresponds to the difference, and relative risk reduction RRR = (*p*_*E*_ − *p*_*C*_)/*p*_*C*_ evaluates the difference in two response rates relative to a reference group. For survival endpoints, the commonly used hazard ratio (HR) is the ratio of the hazard functions for the treatment versus control groups, and ARR characterizes the difference of survival probabilities at a particular time point between two groups. The definitions of RR, ARR, RRR and HR may not be transparent to patients and clinicians. For better understanding of treatment effect, the number of patients needed to treat (NNT), defined as the reciprocal of the difference between *p*_*E*_ and *p*_*C*_, i.e., NNT = 1/(*p*_*E*_ − *p*_*C*_), has been widely advocated for reporting the results of RCTs [2–5]. It can be interpreted as the expected number of patients needed to treat in order to gain one extra response or save one extra life (if mortality is the endpoint) using the treatment vs control. The NNT can be further classified as NNTB (benefit) or NNTH (harm) depending on the beneficial or harmful effects of the treatment [3, 4].

A similar NNT definition has been established for the survival endpoint using ARR. Let *S*_*E*_(*τ*) and *S*_*C*_(*τ*) be the survival probabilities at time *τ* for the experimental and control arms respectively, and then ARR = *S*_*E*_(*τ*) – *S*_*C*_(*τ*), which can be estimated as the difference in the Kaplan-Meier (KM) survival rates at time *τ* [4]. As a result, NNT_surv_ is defined as

$$ {\mathrm{NNT}}_{\mathrm{surv}}=\frac{1}{S_E\left(\tau \right)-{S}_C\left(\tau \right)}, $$

which represents the number of patients needed to treat to prevent one event of interest (e.g., death or disease progression) up to follow-up time *τ*. When the two survival rates are very close at some time points but different at others, NNT_surv_ would vary dramatically over time and sometimes can take very large numbers.

Instead of using the truncated information such as survival rates at a particular time point, one can compute the mean survival (or event-free) time during a prespecified follow-up period, which is known as the restricted mean survival time (RMST) [6–11]. The RMST has been advocated broadly in medical literature as a robust quantification of the treatment effect [8–10]. Due to censoring, the mean survival time is not estimable, while the RMST can be estimated by the area under the KM curve up to a specific time point. Along a similar line, the RMST-based NNT may serve as an alternative to NNT_surv_ for survival endpoints, namely NNT_RMST_, which inherits the advantages of the transparency and unambiguity of RMST in quantifying time-to-event data [12].

The NNT is calculated by first obtaining the difference of clinical measures of interest for two treatments, e.g., response rates, survival rates, or RMSTs, and then taking the reciprocal of the difference. However, without a baseline as the reference, NNT (similar to HR) ignores the absolute scale of the clinical measures and thus may cause ambiguity in certain cases [13–15]. For example, the following two scenarios cannot be distinguished by NNT: (1) *p*_*E*_ = 0.2, *p*_*C*_ = 0.1 and thus NNT = 1/(*p*_*E*_ − *p*_*C*_) = 10; (2) *p*_*E*_ = 0.5 and *p*_*C*_ = 0.4, also leading to NNT = 10. Nevertheless, the two situations are clearly different: the treatment doubles the response rate of the control in the former case while the increment is only 25% in the latter case. The NNT only depends on the difference but not the response rates themselves. Moreover, because ARR is the difference of two probabilities with a range from − 1 to 1, the range of NNT is (−∞, −1] ∪ [1, +∞) rather than the whole real line. If the two response rates are close, NNT takes a very large value and even becomes infinity if the two response rates are exactly the same. When the difference of the two response rates is insignificant, i.e., the corresponding confidence interval (CI) [ARR_lower_, ARR_upper_] of ARR covers zero, the CI of NNT would have a strange form of [NNTB 1/ARR_upper_ to ∞ to NNTH -1/ARR_lower_] [3], which contains infinity in the middle of two numbers. Such an irregular form of CI often causes confusion for clinicians and patients.

To resolve the limitations of NNT with binary and time-to-event endpoints, we propose an alternative quantity, the reduction in the number of patients to treat (RNT), which is computed as the difference of the reciprocals of clinical measures between two arms, i.e., first taking reciprocals of response rates and then obtaining the difference of reciprocals. Unlike NNT which can be infinity if the two response rates are equal, RNT takes a value on the entire real line and its CI always has a regular form, rather than the CI of NNT as the union of two separate intervals when the response rate difference is insignificant.

## Methods

For binary endpoints, we propose a new quantity,

$$ \mathrm{RNT}=1/{p}_C-1/{p}_E, $$

where 1/*p*_*C*_ and 1/*p*_*E*_ are the expected number of patients needed to treat in order to observe one response in the control and experimental groups, respectively. By definition, RNT is computed by first taking reciprocals of the response rates and then obtaining the difference. The RNT can be interpreted as the expected reduction in the number of patients to treat for the treatment compared with the control to induce one response. For the two scenarios considered earlier, RNT equals 5 for the low response case and 0.5 for the high response case, which clearly distinguishes the two situations. Table 1 illustrates the difference between RNT and NNT, where the former depends on individual response rates but the latter does not. Compared with NNT, RNT has a baseline and thus delivers more information on treatment effect. To see connections between different quantities, we can rewrite RNT as

$$ \mathrm{RNT}=\frac{p_E-{p}_C}{p_E{p}_C}=\frac{1}{p_E{p}_C}\frac{1}{\mathrm{NNT}}=\frac{1}{p_E}\mathrm{RRR}. $$

**Table 1:** RNT and NNT under different baseline response rates and response rate differences

Response rate difference	RNT							NNT
	Baseline response rate (control)							
	0.8	0.6	0.4	0.2	0.1	0.05	0.01	
0.2	0.25	0.42	0.83	2.50	6.67	16.00	95.24	5.00
0.15	0.20	0.33	0.68	2.14	6.00	15.00	93.75	6.67
0.1	0.14	0.24	0.50	1.67	5.00	13.33	90.91	10.00
0.05	0.07	0.13	0.28	1.00	3.33	10.00	83.33	20.00
0.01	0.02	0.03	0.06	0.24	0.91	3.33	50.00	100.00
0	0	0	0	0	0	0	0	∞

Not only does RNT involve NNT, but it also includes the multiplication of response rates of both experimental and control arms. Furthermore, it can be rewritten as RRR divided by the response rate of the experimental arm. Through the normal approximation and delta method [16], the two-sided Wald-type 100(1 − *α*)% CI of RNT has the form of

$$ \mathrm{RNT}\pm {z}_{1-\frac{\alpha }{2}}\sqrt{\frac{1-{p}_C}{n_{\mathrm{C}}{p}_C^3}+\frac{1-{p}_E}{n_E{p}_E^3}}, $$

where *n*_*i*_ is the number of patients in arm *i* (*i* = *E*, *C*) and *z*_1 − *α*/2_ is the (1 − *α*/2)-th quantile of the standard normal distribution. When sample size is small, the Wald CIs may not be accurate, and instead the bootstrap percentile CIs or exact CIs can be used. Detailed procedures of constructing bootstrap percentile CIs [17] and exact CIs for RNT are presented in Supplementary Materials and numerical studies were conducted to examine the performances of various CIs (see Supplementary Table S1).

For survival endpoints, similar to NNT_surv_, we can define RNT_surv_ based on the survival rates of two arms,

$$ {\mathrm{RNT}}_{\mathrm{surv}}\left(\tau \right)=1/{S}_C\left(\tau \right)-1/{S}_E\left(\tau \right), $$

where τ is a pre-specified follow-up time. The RNT_surv_ can be interpreted as the expected reduction in the number of patients to treat in the experimental arm compared with the control arm to prevent one adverse event (e.g., death or disease progression) up to time *τ*. On the other hand, the difference in RMSTs represents the average gain in survival time for patients receiving the treatment in comparison with the control during the *τ*-period follow-up. The definition of RMST-based RNT is

$$ \mathrm{RN}{\mathrm{T}}_{\mathrm{RMST}}\left(\tau \right)=\frac{1}{{\mathrm{RMST}}_C\left(\tau \right)/\tau }-\frac{1}{{\mathrm{RMST}}_E\left(\tau \right)/\tau }=\tau \left(\frac{1}{{\mathrm{RMST}}_C\left(\tau \right)}-\frac{1}{{\mathrm{RMST}}_E\left(\tau \right)}\right), $$

where RMST_*E*_(*τ*) and RMST_*C*_(*τ*) are the RMSTs up to time *τ* in the experimental and control arms, respectively. The RNT_RMST_ quantifies the reduction in the number of patients to treat in the experimental arm compared with the control arm in order to obtain one survival case by time *τ*, which is equivalent to obtaining a total of *τ* event-free survival time. In Sections 3 and 4, we compare RNT_RMST_ with an RMST-based NNT [12],

$$ {\mathrm{NNT}}_{\mathrm{RMST}}\left(\tau \right)=\frac{\tau }{{\mathrm{RMST}}_E\left(\tau \right)-{\mathrm{RMST}}_C\left(\tau \right)}, $$

which can be interpreted as the number needed to treat in the experimental arm compared with the control arm in order to obtain one extra survival case by time *τ* or gain a total of *τ* event-free survival time.

To quantify the uncertainty of RNT_surv_ and RNT_RMST_, we can compute standard errors by the delta method and construct Wald CIs by normal approximation [16]. The corresponding 100(1 − *α*)% CI for RNT_surv_ and RNT_RMST_ can be calculated as

$$ \mathrm{RN}{\mathrm{T}}_{\mathrm{surv}}\left(\tau \right)\pm {z}_{1-\frac{\alpha }{2}}\sqrt{\frac{\mathrm{Var}\left({S}_C\left(\tau \right)\right)}{S_C^4\left(\tau \right)}+\frac{\mathrm{Var}\left({S}_E\left(\tau \right)\right)}{S_E^4\left(\tau \right)}}, $$

$$ \mathrm{RN}{\mathrm{T}}_{\mathrm{RMST}}\left(\tau \right)\pm \tau {z}_{1-\frac{\alpha }{2}}\sqrt{\frac{\mathrm{Var}\left({\mathrm{RMST}}_C\left(\tau \right)\right)}{{\left({\mathrm{RMST}}_C\left(\tau \right)\right)}^4}+\frac{\mathrm{Var}\left({\mathrm{RMST}}_E\left(\tau \right)\right)}{{\left({\mathrm{RMST}}_E\left(\tau \right)\right)}^4}}, $$

where Var(.) represents the variance of the survival rate or RMST [11, 18]. Similar to binary cases, the Wald CI may not be accurate with small sample size, while a percentile CI obtained from a perturbation-resampling approach [11] can be used as an alternative. Supplementary Materials contain detailed steps to construct the CIs of RNT based on survival rates and RMSTs via the perturbation-resampling method as well as simulation studies to compare their performances (see Supplementary Table S2).

## Results

We demonstrate the advantages of our proposed RNT over NNT in five real trials, including two trials with binary endpoints and three trials with time-to-event endpoints. Wald CIs are used for binary data and perturbation-resampling CIs are used for survival data as suggested by the simulations in Supplementary Materials.

### Example 1: KCSG-LU05–04 trial and GILT trial

In cancer research, the commonly used overall response rate (ORR) is defined as the proportion of patients whose tumours are no longer detectable (complete response) or the tumour size has significantly decreased (partial response) after treatment. For inoperable stage III non-small-cell lung cancer (NSCLC), two clinical trials [19, 20] were conducted to examine the efficacy of concurrent chemotherapy alone (CRT) versus concurrent chemotherapy plus consolidation (CRT-C).

In the KCSG-LU05–04 trial [19], 420 patients were randomized with 211 in the CRT arm and 209 in the CRT-C arm. Responses to therapy were observed on 81 patients treated with CRT and 90 with CRT-C. The ORR was 38.4% for CRT and 43.1% for CRT-C, leading to NNT 21.4 (95% CI [NNTB 7.1 to ∞ to NNTH 21.2]). Thus, the average number of patients needed to treat using CRT-C compared with CRT in order to obtain one extra response was 21.4.

Flentje et al. [20] conducted a similar trial, named GILT, to compare the CRT alone versus CRT plus consolidation, with 105 patients enrolled in the CRT arm and 96 in the CRT-C arm. The ORR was 24.8 and 29.1% for CRT and CRT-C respectively, which led to NNT 22.7 (95% CI [NNTB 6.0 to ∞ to NNTH 12.7]), similar to the NNT in the previous trial.

Although the two NNTs of the aforementioned NSCLC trials were close and thus represented similar benefit of the additional consolidation therapy, there was substantial difference in the ORR of the CRT arm between the two trials (38.4% versus 24.8%). The NNT is calculated as the reciprocal of the absolute difference and thus fails to convey the information on the response rates themselves. In contrast, RNT involves a baseline when calculating the difference of the reciprocals of the response rates of two arms. The estimated RNT for the KCSG-LU05–04 trial was 0.28 (95% CI [− 0.29, 0.86]), while that for the GILT trial was 0.61 (95% CI [− 1.11, 2.33]). The two RNTs are very different, and the latter is more than double of the former. Compared with CRT, on average 0.28 fewer patient would be needed by treatment CRT-C to obtain one response for the KCSG-LU05–04 trial, and that for the GILT trial was 0.61. Moreover, the CIs of RNT have the standard form, rather than the irregular form with the infinity in the range of NNTB and NNTH under the NNT formulation.

### Example 2: S0226 trial

**Fig. 1:**
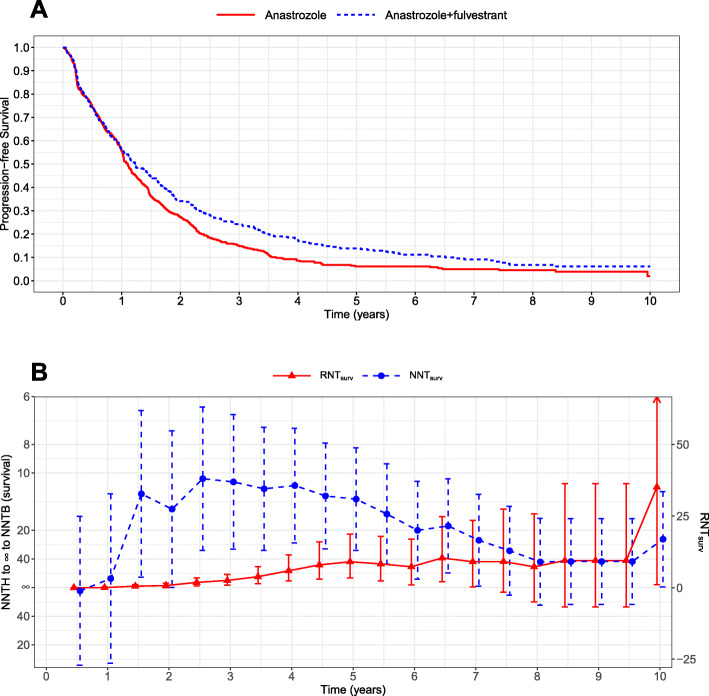
NNT vs RNT with perturbation-resampling CIs for the S0226 trial [21]. **a** Kaplan-Meier estimates of progression-free survival curves for the fulvestrant plus anastrozole therapy and anastrozole therapy alone; **b** NNTs and RNTs calculated from survival rates with the 95% CIs

The S0226 trial [21] was a multi-center, randomized, open-label study with patients of metastatic breast cancer to evaluate the potential benefit of adding fulvestrant to anastrozole therapy versus anastrozole alone. A total of 694 patients were enrolled with 345 assigned to anastrozole alone and 349 to fulvestrant plus anastrozole therapy. The primary endpoint was progression-free survival (PFS) and the corresponding Kaplan-Meier curves are shown in Fig. 1a where the two survival curves are nearly overlapped during the first year and then separated afterwards, but finally almost converge toward the end of the study. We reconstructed the data from the PFS curves for all eligible patients [22]. The estimates of NNT_surv_ and RNT_surv_ together with their CIs at different time points during the 10-year follow-up period are shown in Fig. 1b and Supplementary Table S3.

As shown in Fig. 1b (noting the difference in the y-axis for NNT and RNT), NNT_surv_ takes extremely large values (e.g., infinity) during the first year follow-up because the two survival curves are almost indistinguishable, and continues to decrease till year 3 and then starts to increase after year 4. In contrast, the values of RNT_surv_ remain quite stable during the entire follow-up except at year 10, where the survival rates of both arms are low and RNT_surv_ becomes sensitive due to the direct inversion of survival rates. The values of NNT_surv_ at years 3 and 4 are close, which fails to deliver the information that the survival rate of the anastrozole alone at year 3 was about twice of that at year 4. Such findings, however, can be revealed by the significant gap between the values of RNT_surv_ at years 3 and 4 as shown in Fig. 1b and Supplementary Table S3. The estimated RNT_surv_ at year 3 was 2.52 (95% CI [0.89, 4.60]) and that at year 4 was 5.89 (95% CI [2.32, 11.42]). Compared with the anastrozole therapy alone, 2.52 and 5.89 fewer patients are needed to treat with the fulvestrant plus anastrozole therapy to obtain one survival case at years 3 and 4, respectively.

### Example 3. Urgent endoscopy vs early endoscopy for acute upper gastrointestinal bleeding

**Fig. 2:**
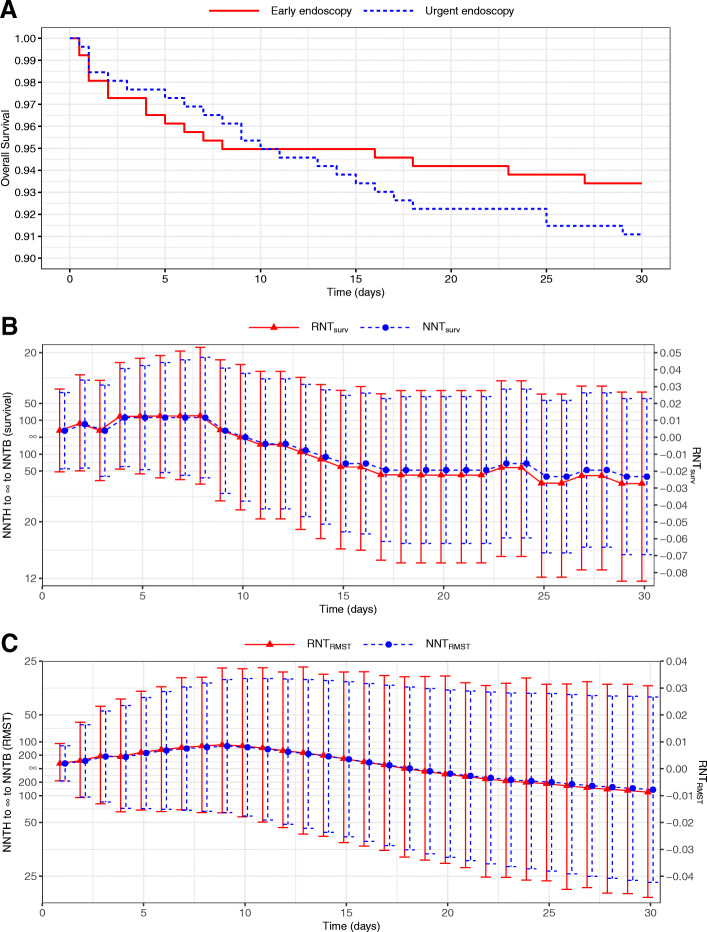
NNT vs RNT with perturbation-resampling CIs from the urgent endoscopy study [23]. **a** Kaplan-Meier estimates of overall survival curves for the urgent endoscopy and early endoscopy groups; **b** NNTs and RNTs calculated from the survival rates with the 95% CIs; **c** NNTs and RNTs calculated from RMSTs with the 95% CIs

A recent randomized clinical trial was conducted by Lau et al. [23] to evaluate clinical performance of urgent endoscopy versus early endoscopy in high-risk patients with upper gastrointestinal bleeding. A total of 516 patients were enrolled and equally randomized to the urgent and early endoscopy groups. The primary endpoint was death from any cause during the 30-day follow-up period and the overall survival (OS) curves are shown in Fig. 2a. The two OS curves cross once at day 10. When the two survival curves make a crossing, the survival probabilities of two arms are equal at the crossing point. As a consequence, the NNT estimate would be infinity for which the clinical meaning is obscure, while the RNT estimate equals 0, indicating no difference in the treatment benefit. The values of NNT_surv_ and RNT_surv_ calculated from the OS probabilities during the 30-day follow-up period are plotted in Fig. 2b. Compared with the irregular y-axis of NNTs (i.e., the left-hand side y-axis of Fig. 2b) ranging from NNTH to ∞ to NNTB, the commonly used axis of RNTs (i.e., the right-hand side y-axis of Fig. 2b) has the zero point at the center. When NNT_surv_ at day 10 is ∞, the corresponding RNT_surv_ is zero.

Urgent endoscopy performed better with a lower death rate up to day 10, while early endoscopy showed more benefit during the rest of the follow-up. At day 11, the value of RNT_surv_ is − 0.004 (95% CI [− 0.048, 0.039]), indicating that the early endoscopy (control) arm performed slightly better on reducing all-cause deaths. However, such an interpretation ignores the fact that the OS rate of the urgent endoscopy group was higher during the first 10 days and the result at a specific time point only includes local information rather than the global treatment effect and thus conveys misleading findings. As an alternative, RMST can be used to assess the entire profile of treatment effect over time, which can serve as the basis for the calculation of the RMST-based NNT and RNT. Fig. 2c displays NNT_RMST_ and RNT_RMST_ from day 1 to day 30. The value of RNT_surv_ depends on the survival probability at each time point, and thus RNT_surv_ fluctuates more drastically over time. In contrast, RNT_RMST_ represents a cumulative summary of survival information up to a specified time point, which changes more smoothly over time. The RNT_RMST_ at day 11 was 0.008 (95% CI [− 0.020, 0.038]), indicating slight superiority for urgent endoscopy. Note that the RNTs based on survival rates and RMSTs have opposite signs, although both are statistically insignificant at the 5% significance level. As shown in Supplementary Table S4, RNT_surv_ at day 30 is − 0.027 (95% CI [− 0.085, 0.027]), i.e., to obtain 100 survival cases by day 30, urgent endoscopy (experimental) needs to treat on average 2.7 more patients compared with early endoscopy (control). The RNT_RMST_ at day 30 is − 0.009 (95% CI [− 0.048, 0.031]), which means that during the 30-day follow-up, on average 0.9 fewer patient would be needed for early endoscopy to obtain 100 survival cases at day 30 (or 30 × 100 = 3000 patient-days), compared with urgent endoscopy.

### Example 4. Prophylactic cranial irradiation trial

**Fig. 3:**
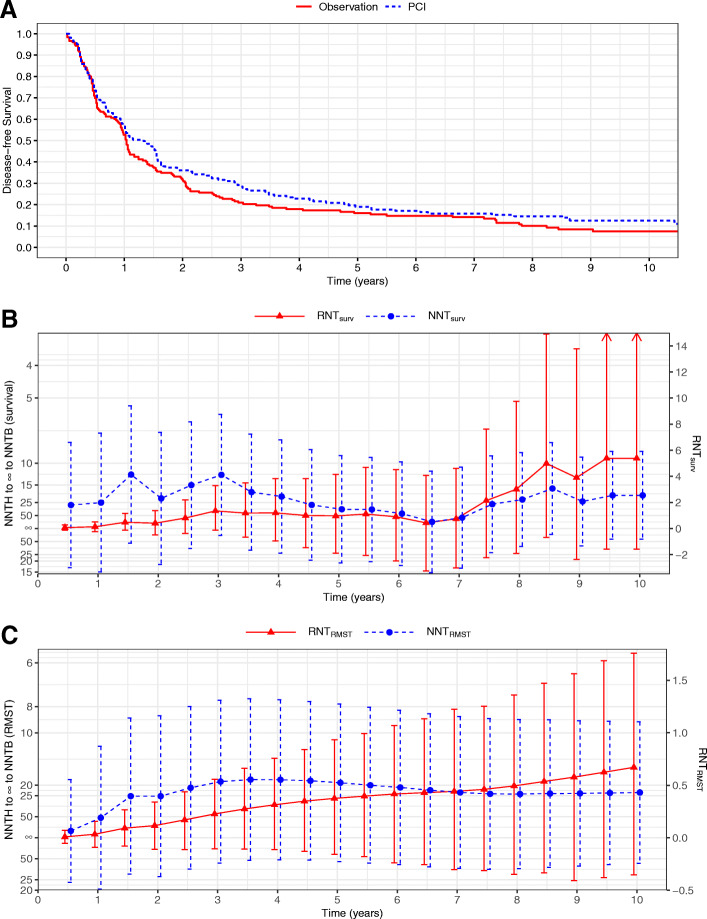
NNT vs RNT with perturbation-resampling CIs for the prophylactic cranial irradiation trial [24]. **a** Kaplan-Meier estimates of disease-free survival curves for the prophylactic cranial irradiation (PCI) and observation groups; **b** NNTs and RNTs calculated from the survival rates with the 95% CIs; **c** NNTs and RNTs calculated from RMSTs with the 95% CIs

The RTOG 0214 trial was a phase 3 randomized study to determine whether prophylactic cranial irradiation (PCI) could improve survival in patients with locally advanced NSCLC compared with the observation group after effective locoregional/systemic therapy [24]. The trial enrolled 340 patients, with 163 randomized to the PCI group and 177 to the observation group. The disease-free survival (DFS) curves of the PCI and observation groups in Fig. 3a are intertwined during the first half year and then diverge and converge several times during the remaining follow-up period. As a result, the estimates of NNT_surv_ fluctuate more dramatically during the first 3 years and in years 6–8, as shown in Fig. 3b and Supplementary Table S6. There are several pairs of time points, at which the values of two NNT_surv_ are close while those of RNT_surv_ are quite different; for example, year 1 versus year 9, year 2 versus year 8, and year 4 versus year 10. The NNT_surv_ at year 4 is 20.42 (95% CI [NNTB 7.37 to ∞ to NNTH 26.44]) and that at year 10 is 19.67 (95% CI [NNTB 8.47 to ∞ to NNTH 60.67]), indicating that the numbers of patients needed for the PCI arm to obtain one more survival case compared with the observation group at years 4 and 10 are both about 20. However, the estimated RNT_surv_ at years 4 and 10 are 1.20 (95% CI [− 0.96, 3.82]) and 5.38 (95% CI [− 1.59, 18.08]), respectively. The value of RNT_surv_ at year 10 is about 4.5 times of that at year 4. Compared with the observation arm, on average the PCI group needs to treat 1.20 and 5.38 fewer patients to obtain one survival case at year 4 and 10, respectively.

As shown in Fig. 3b and Supplementary Table S7, RNT_RMST_ continues to increase over years, and its value at year 10 is 0.67 (95% CI [− 0.35, 1.76]). Compared with the observation arm, the PCI arm needs to treat 0.67 fewer patient to obtain one disease-free patient during the 10-year follow-up period. The NNT_RMST_ exhibits a similar but more smoothed trend compared with NNT_surv_, which decreases in the first 3 years and then increases and finally drops again during the later follow-up of the study. At years 7 and 10, the corresponding estimates of NNT_RMST_ are very close, 23.19 and 23.21, which cannot discriminate the treatment benefit. While the RNT_RMST_ at year 10 is about 1.5 times of that at year 7, which not only conveys the absolute RMST difference but also the information on the values of RMSTs for the experimental and control arms.

## Hypothetical examples

For better illustration, we further use hypothetical examples to discuss the advantages and disadvantages of RNT in comparison with NNT under binary and survival endpoints. Table 1 shows the values of NNT and RNT under various baseline response rates and response rate differences. With a fixed response rate difference, the NNT remains the same, while there is an obvious reduction in RNT as the baseline rate increases. For example, when ARR = 0.1, NNT is 10 regardless of the value of the baseline response rate; however, RNT ranges from 0.14 to 90.9 when the baseline response rate decreases from 0.8 to 0.01. More importantly, NNT would be infinity when the response rate difference is zero, which is difficult to interpret in comparison with the corresponding value of zero for RNT. Due to the definition as the difference of the reciprocals of response rates, RNT is sensitive to the change of the response rate difference when the baseline response rate is low. When the response rates are high (e.g., when the baseline response rate is greater than 0.6), the value of RNT tends to be small and sometime can be less than one.

**Fig. 4:**
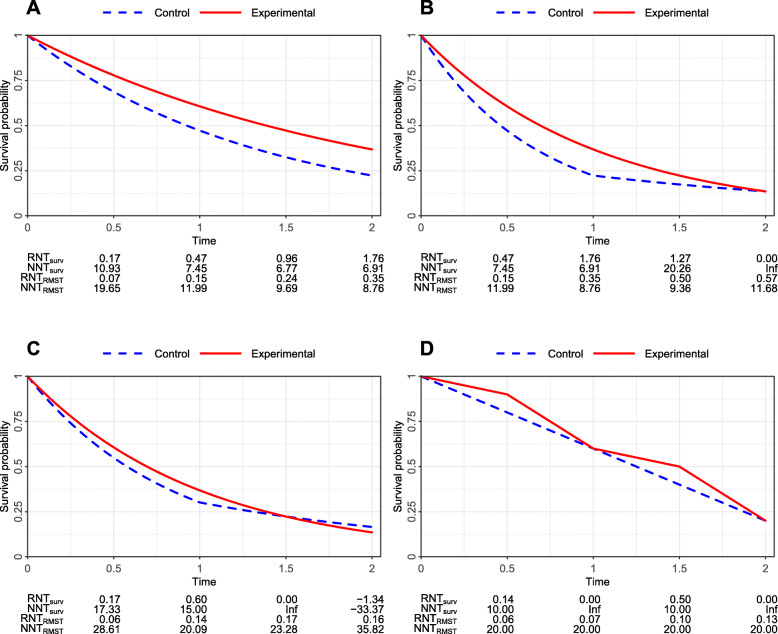
Hypothetical examples under four survival scenarios. **a** Proportional hazards; **b** Survival curves cross at the end of the follow-up period; **c** Survival curves cross during the follow-up period; **d** Survival curves cross during and at the end of the follow-up period

Four hypothetical scenarios are constructed to compare NNT and RNT based on survival rates and RMSTs, respectively. Scenario 1 (Fig. 4a) reflects the proportional hazards case where the experimental arm is consistently better than the control arm. The decreasing trend of NNT_surv_ and NNT_RMST_ and increasing trend of RNT_surv_ and RNT_RMST_ at four time points demonstrate an increasing treatment difference over the follow-up period. Compared with NNT_surv_ and NNT_RMST_, relatively larger changes can be observed for RNT_surv_ and RNT_RMST_ from time points 1 to 2. In Scenario 2 (Fig. 4b), the two survival curves diverge during the first half of the follow-up and then converge in the second half. The value of NNT_surv_ is infinity at the end of the study, for which the clinical interpretation is not easy. In contrast, RNT_surv_ takes a value of zero at time point 2, clearly indicating no treatment difference because the same number of patients is needed to treat in order to obtain one survival case at time point 2 for the two arms. However, since the survival rate at a particular time point can only reflect the local survival information, NNT_surv_ and RNT_surv_ fail to capture the divergence and convergence pattern of survival curves. In such cases, NNT_RMST_ and RNT_RMST_ at the end of the follow-up can quantify the entire profile of the two survival curves. In Scenario 3, we consider another crossing survival case where the two survival curves intersect at time point 1.5 during the follow-up period. The values of NNT_surv_ and RNT_surv_ show opposite signs before and after the crossing point of survival curves, ignoring the fact that the survival curve of the experimental arm is above that of the control arm till time point 1.5. In contrast, NNT_RMST_ and RNT_RMST_ successfully convey such information for which the positive values reflect the favor of the experimental arm. Scenario 4 shows that two survival curves diverge and converge twice during the follow-up period. The NNT_surv_ has the same value of 10 at time points 0.5 and 1.5, while the values of RNT_surv_ at the two time points are very different. Similarly, NNT_RMST_ takes the same value of 20 at all four time points, while different values of RNT_RMST_ can reflect the change in the baseline RMST.

## Discussion

As an essential component of RCTs, interpreting the evidence of the treatment effect to practitioners plays a vital role in their decision making under the risk-benefit consideration. In binary data cases, the popularity of ARR in medical research makes NNT a primary tool for quantifying and presenting treatment effect. However, as the reciprocal of ARR, NNT fails to convey information on the absolute scale of the response rates and its irregular form of CI containing the infinity between the lower and upper bounds often causes confusion. Similar issues also arise for survival endpoints when using survival rates at a particular time point.

As an alternative, the proposed RNT reflects both the difference and absolute values of the clinical measurement of interest, and the corresponding CI always has the regular form with the center around 0 when the two clinical measurements are close or equal, leading to a more transparent presentation on the variation of the treatment difference. Moreover, when conducting meta-analysis by pooling information from multiple RCTs, the pooled NNT could be misleading and the irregular CIs would be difficult to be used in conjunction with regular CIs [25, 26]. In contrast, the pooled RNT using the regular form of CI can still maintain its statistical properties and clinically meaningful interpretation.

Although the proposed RNT has attractive features on the quantification of clinical benefit, there exist several limitations. First, when the two clinical measurements are close to each other or when both take large values, RNT would have a small value. For example, if the response rates are 85 and 80% for the experimental and control arms respectively, RNT is equal to 0.074, i.e., on average 0.074 fewer patient would be needed by the experimental treatment to obtain one response compared with the control. In such cases, one can change the unit of the response from one to 100, i.e., on average 7.4 fewer patients are needed by the experimental treatment to obtain 100 responses compared with the control. In addition, similar to NNT, RNT is directly computed from the clinical quantities (e.g., response rates, survival rates and RMSTs) and thus all versions of RNTs share the limitations of NNT [13–15]. The values of RNT may not be comparable when the evaluated clinical endpoints are different, e.g., one cannot aggregate RNTs obtained from overall survival and progression-free survival. Moreover, RNT works for binary and time-to-event endpoints, but not for continuous endpoints. Focusing on the summary data rather than individual-level patient data, RNT evaluates the expectation for all patients in a clinical trial rather than characterizing individual distinctions.

## Conclusion

Despite the limitations, RNT is a metric of great value and has advantages over the commonly used NNT. It can help clinicians and patients understand treatment benefits and their variations from a clinically clear and intuitive perspective.

## Supplementary Information

**Additional file 1.**

## Data Availability

The individual-level patient data of the real trials used and analyzed in this manuscript were reconstructed from the corresponding Kaplan-Meier survival curves and are available from the authors upon request. Public access to these five real randomized controlled trials can be found in their references. KCSG-LU05–04 trial (Example 1): Ahn JS, Ahn YC, Kim JH, et al. Multinational randomized phase III trial with or without consolidation chemotherapy using docetaxel and cisplatin after concurrent chemoradiation in inoperable stage III non–small-cell lung cancer: KCSG-LU05–04. J Clin Oncol. 2015;33:2660–2666. GILT trial (Example 1): Flentje M, Huber RM, Engel-Riedel W, et al. GILT—A randomised phase III study of oral vinorelbine and cisplatin with concomitant radiotherapy followed by either consolidation therapy with oral vinorelbine and cisplatin or best supportive care alone in stage III non-small cell lung cancer. Strahlenther Onko. 2016;192:216–222. S0226 trial (Example 2): Mehta RS, Barlow WE, Albain KS, et al. Overall survival with fulvestrant plus anastrozole in metastatic breast cancer. N Engl J Med. 2019;380:1226–1234. Urgent endoscopy vs early endoscopy for acute upper gastrointestinal bleeding (Example 3): Lau JY, Yu Y, Tang RS, et al. Timing of endoscopy for acute upper gastrointestinal bleeding. New England Journal of Medicine. 2020;382:1299–1308. Prophylactic cranial irradiation trial (Example 4): Sun A, Hu C, Wong SJ, et al. Prophylactic cranial irradiation vs observation in patients with locally advanced non–small cell lung cancer: a long-term update of the NRG Oncology/RTOG 0214 phase 3 randomized clinical trial. JAMA Oncology. 2019;5:847–855.

## Abbreviations

ARR: Absolute risk reduction
DFS: Disease-free survival
CRT: Concurrent chemotherapy
CRT-C: Concurrent chemotherapy plus consolidation
CI: Confidence interval
HR: Hazard ratio
KM: Kaplan-Meier
NNT: Number needed to treat
NNTB: Number needed to treat to benefit
NNTH: Number needed to treat to harm
RNT: Reduction in number to treat
NSCLC: Non-small-cell lung cancer
ORR: Overall response rate
OS: Overall survival
PCI: Prophylactic cranial irradiation
PFS: Progression-free survival
RCT: Randomized controlled trials
RMST: Restricted mean survival time
RR: Relative risk
RRR: Relative risk reduction
