# Supplementary material for: Reduction in number to treat versus number needed to treat
Source: BMC Med Res Methodol. 2021 Mar 9;21:48. doi: 10.1186/s12874-021-01246-5 (PMC7945324; doi:10.1186/s12874-021-01246-5)
Supplement: Supplementary file 1 — Additional file 1. [file 12874_2021_1246_MOESM1_ESM.docx]

**Supplementary Materials of “Reduction in Number to Treat versus Number Needed to Treat”**

# **Reduction in the number of patients to treat (RNT) based on binary endpoints**

For randomized controlled trials (RCTs) that evaluate the response rates of the experimental treatment and the control based on binary endpoints, $\mathrm{RNT}$ is estimated as the difference of the reciprocals of the estimated response rates for the control and experimental arms,

$$\hat{\mathrm{RNT}}=\frac{1}{\hat{p}_{C}}-\frac{1}{\hat{p}_{E}},$$

where $\hat{p}_{E}$ and $\hat{p}_{C}$ are the estimated response rates for the experimental and control groups respectively. For the special case with $\hat{p}_{C}=\hat{p}_{E}=0,$ we define $\hat{\mathrm{RNT}}=0$.

Given the significance level $\alpha$, we can construct the $100\left( 1-\alpha\right)\%$ Wald confidence interval (CI) of $\hat{\mathrm{RNT}}$ by normal approximation [1],

$$\hat{\mathrm{RNT}}\pm z_{1-\frac{\alpha}{2}}\mathrm{SE}\left( \hat{\mathrm{RNT}} \right),$$

where $z_{1-\alpha/2}$ is the $(1-\alpha/2)$th quantile of the standard normal distribution and

$$\mathrm{SE}\left( \hat{\mathrm{RNT}} \right)=\sqrt{\mathrm{Var}\left( \frac{1}{\hat{p}_{C}} \right)+\mathrm{Var}\left( \frac{1}{\hat{p}_{E}} \right)}.$$

Using the delta method [1], we can calculate the variance of the reciprocal of the estimated response rates as follows,

$$\mathrm{Var}\left( \frac{1}{\hat{p}_{i}} \right)=\frac{1}{\hat{p}_{i}^{4}}\mathrm{Var}\left( \hat{p}_{i} \right)=\frac{1-\hat{p}_{i}}{n_{i}\hat{p}_{i}^{3}}, i=E,C,$$

where $n_{i}$ is the number of patients in arm $i$. The $100\left( 1-\alpha\right)\%$ CI has the form,

$$\hat{\mathrm{RNT}}\pm z_{1-\frac{\alpha}{2}}\sqrt{\frac{1-\hat{p}_{C}}{n_{C}\hat{p}_{C}^{3}}+\frac{1-\hat{p}_{E}}{n_{E}\hat{p}_{E}^{3}}}.$$

Moreover, the CI of $\mathrm{RNT}$ can be used in the hypothesis testing with

$H_{0}:RNT=0 vs H_{1}:RNT\neq0$.

The null hypothesis would be rejected at the significance level $\alpha$ if the $100\left( 1-\alpha\right)\%$ CI covers zero. When the two response rates are close, the value of $\mathrm{RNT}$ would be close to zero, unlike the commonly used NNT that might yield a very large value and an unusual CI containing $\infty.$

However, the normal approximation and delta method might not work well with small sample size. When sample size is small, the exact confidence interval can be calculated for better accuracy and coverage probability. Given the numbers of responses $\left( y_{E},y_{C} \right)$ and the total numbers of patients $\left( n_{E},n_{C} \right)$ in the treatment and control arms, we can calculate the Clopper-Pearson exact interval [1] of $\left( \hat{p}_{C},\hat{p}_{E} \right)$ by solving the equations,

$$\sum_{k=y_{i}}^{n_{i}} \binom{n_{i}}{k} p_{L,i}^{k}\left( 1-p_{L,i} \right)^{n_{i}-k}=\frac{\alpha}{2}, i=E,C,$$

$$\sum_{k=0}^{y_{i}} \binom{n_{i}}{k} p_{U,i}^{k}\left( 1-p_{U,i} \right)^{n_{i}-k}=\frac{\alpha}{2}, i=E,C,$$

where $p_{L,i}$ and $p_{U,i}$ denote the lower and upper limits of  $\hat{p}_{i}$ at the significance level $\alpha$ for arm $i$, respectively. By inverting the distribution function of a Beta distribution, we can obtain

$$p_{L,i}(\alpha)=\left\{ \begin{aligned} F_{\mathrm{Beta}}^{-1}\left( \frac{\alpha}{2};y_{i},n_{i}-y_{i}+1 \right), &1\leq y_{i}\leq n_{i}-1, \\ 0, &y_{i}=0, \\ \left( \frac{\alpha}{2} \right)^{\frac{1}{n}}, &y_{i}=n_{i}, \end{aligned} \mathrm{for} i=E,C, \right.$$

$$p_{U,i}(\alpha)=\left\{ \begin{aligned} F_{\mathrm{Beta}}^{-1}\left( 1-\frac{\alpha}{2};y_{i}+1,n_{i}-y_{i} \right), &1\leq y_{i}\leq n_{i}-1, \\ 1-\left( \frac{\alpha}{2} \right)^{\frac{1}{n}}, &y_{i}=0, \\ 1, &y_{i}=n_{i}, \end{aligned} \mathrm{for} i=E,C, \right.$$

where $F_{\mathrm{Beta}}\left( \cdot;a,b \right)$ is the cumulative density function of a Beta distribution with parameters $\left( a,b \right).$ As a result, we can calculate the exact CIs of $\hat{p}_{C}$ and $\hat{p}_{E}$ under any confidence level and draw samples by treating $p_{L,i}$ and $p_{U,i}$ as quantile functions. The exact CI of $\hat{\mathrm{RNT}}$ given the observed data$\left( y_{E},y_{C} \right)$ and $\left( n_{E},n_{C} \right)$ can be approximated by the following steps.

1. For the $j$-th simulation, $j=1,\ldots,B,$sample $v_{i}^{\left( j \right)}\sim\mathrm{Unif}\left( 0,1 \right)$ for $i=E,C.$
2. $p_{i}^{(j)}=\left\{ \begin{aligned} p_{L,i}\left( 2v_{i}^{\left( j \right)} \right), &v_{i}^{\left( j \right)}\leq0.5, \\ p_{U,i}\left( {2v}_{i}^{\left( j \right)}-1 \right), &v_{i}^{\left( j \right)}>0.5, \end{aligned} \right.$ for $i=E,C.$
3. $\mathrm{RN}T_{\mathrm{exact}}^{(j)}=\frac{1}{p_{C}^{(j)}}-\frac{1}{p_{E}^{\left( j \right)}}$.
4. The estimated lower and upper bounds of $\hat{\mathrm{RNT}}$ can be approximated by the $\left( \frac{\alpha}{2} \right)$-th and $\left( 1-\frac{\alpha}{2} \right)$-th empirical quantiles of $\left\{ \mathrm{RN}T_{\mathrm{exact}}^{(j)},j=1,\ldots,B \right\}$.

Another way is to use the bootstrap percentile CI, which can be constructed as follows.

1. Obtain $\hat{p}_{E}=\frac{y_{E}}{n_{E}}, \hat{p}_{C}=\frac{y_{C}}{n_{C}}$.
2. For the $j$-th simulation, $j=1,\ldots,B$, sample $\tilde{y}_{E}^{(j)}\sim\mathrm{Binomial}\left( \hat{p}_{E}, n_{E} \right), \tilde{y}_{C}^{(j)}\sim\mathrm{Binomial}\left( \hat{p}_{C}, n_{C} \right)$.
3. Compute $\mathrm{RN}T_{\mathrm{boot}}^{(j)}=\frac{1}{\tilde{y}_{E}^{\left( j \right)}/n_{E}}-\frac{1}{\tilde{y}_{C}^{\left( j \right)}/n_{C}}$.
4. The estimated lower and upper bounds of $\hat{\mathrm{RNT}}$ can be approximated by the $\left( \frac{\alpha}{2} \right)$-th and $\left( 1-\frac{\alpha}{2} \right)$-th empirical quantiles of $\left\{ \mathrm{RN}T_{\mathrm{boot}}^{(j)},j=1,\ldots,B \right\}$.

Simulation studies were conducted to examine the performances of the Wald, bootstrap percentile and exact CIs of RNT for binary data cases under various settings of sample size, response rates in the experimental and control arms. Supplementary Table S1 presents the binary simulation results with a confidence level of 95%.

**Table S1. Binary endpoint simulation results for coverage probabilities of the Wald, bootstrap and exact confidence intervals of RNT.**

| $n$ | $p_{E}$ | $p_{C}$ | CP (Wald) | CP (Bootstrap) | CP (Exact) |
| --- | --- | --- | --- | --- | --- |
| 20 | 0.1 | 0.2 | 0.835 | 0.903 | 0.982 |
|  | 0.2 | 0.2 | 0.975 | 0.949 | 0.981 |
|  | 0.3 | 0.2 | 0.980 | 0.933 | 0.975 |
|  | 0.3 | 0.4 | 0.990 | 0.940 | 0.974 |
|  | 0.4 | 0.4 | 1.000 | 0.956 | 0.970 |
|  | 0.5 | 0.4 | 0.989 | 0.941 | 0.970 |
|  | 0.5 | 0.6 | 0.985 | 0.940 | 0.972 |
|  | 0.6 | 0.6 | 0.998 | 0.956 | 0.972 |
|  | 0.7 | 0.6 | 0.983 | 0.939 | 0.972 |
|  | 0.7 | 0.8 | 0.976 | 0.939 | 0.980 |
|  | 0.8 | 0.8 | 0.992 | 0.949 | 0.981 |
|  | 0.9 | 0.8 | 0.949 | 0.923 | 0.981 |
| 100 | 0.1 | 0.2 | 0.947 | 0.944 | 0.965 |
|  | 0.2 | 0.2 | 0.990 | 0.953 | 0.963 |
|  | 0.3 | 0.2 | 0.969 | 0.947 | 0.964 |
|  | 0.3 | 0.4 | 0.968 | 0.947 | 0.962 |
|  | 0.4 | 0.4 | 0.969 | 0.954 | 0.963 |
|  | 0.5 | 0.4 | 0.962 | 0.942 | 0.958 |
|  | 0.5 | 0.6 | 0.968 | 0.954 | 0.969 |
|  | 0.6 | 0.6 | 0.964 | 0.955 | 0.963 |
|  | 0.7 | 0.6 | 0.961 | 0.949 | 0.964 |
|  | 0.7 | 0.8 | 0.959 | 0.947 | 0.966 |
|  | 0.8 | 0.8 | 0.954 | 0.953 | 0.962 |
|  | 0.9 | 0.8 | 0.953 | 0.944 | 0.961 |
| 200 | 0.1 | 0.2 | 0.953 | 0.947 | 0.962 |
|  | 0.2 | 0.2 | 0.972 | 0.956 | 0.964 |
|  | 0.3 | 0.2 | 0.958 | 0.944 | 0.958 |
|  | 0.3 | 0.4 | 0.958 | 0.947 | 0.959 |
|  | 0.4 | 0.4 | 0.964 | 0.956 | 0.962 |
|  | 0.5 | 0.4 | 0.958 | 0.947 | 0.959 |
|  | 0.5 | 0.6 | 0.954 | 0.946 | 0.958 |
|  | 0.6 | 0.6 | 0.956 | 0.956 | 0.961 |
|  | 0.7 | 0.6 | 0.958 | 0.952 | 0.962 |
|  | 0.7 | 0.8 | 0.952 | 0.945 | 0.958 |
|  | 0.8 | 0.8 | 0.955 | 0.956 | 0.964 |
|  | 0.9 | 0.8 | 0.951 | 0.946 | 0.960 |
| 500 | 0.1 | 0.2 | 0.951 | 0.946 | 0.954 |
|  | 0.2 | 0.2 | 0.960 | 0.954 | 0.956 |
|  | 0.3 | 0.2 | 0.952 | 0.944 | 0.953 |
|  | 0.3 | 0.4 | 0.951 | 0.949 | 0.955 |
|  | 0.4 | 0.4 | 0.955 | 0.954 | 0.957 |
|  | 0.5 | 0.4 | 0.953 | 0.952 | 0.957 |
|  | 0.5 | 0.6 | 0.951 | 0.950 | 0.955 |
|  | 0.6 | 0.6 | 0.955 | 0.954 | 0.957 |
|  | 0.7 | 0.6 | 0.950 | 0.947 | 0.953 |
|  | 0.7 | 0.8 | 0.950 | 0.947 | 0.954 |
|  | 0.8 | 0.8 | 0.953 | 0.954 | 0.958 |
|  | 0.9 | 0.8 | 0.948 | 0.946 | 0.956 |

CP represents coverage probability; $n$ is the sample size; $p_{E}$ and $p_{C}$ are the true response rates for the experimental and control groups, respectively.

When sample size is large ($n=500$), all the three types of CIs perform well with the coverage probabilities (CPs) close to the nominal level 95%. With small ($n=20$) or moderate (n=100) sample size, the exact CI might be too conservative, especially for small sample size (CPs over 97%). The bootstrap percentile approach can provide appropriate interval estimates for RNT, except for the scenario with small sample size ($n=20)$ and extreme event rates for the experimental and control arms ($p_{E}=0.1, p_{C}=0.2; p_{E}=0.9, p_{c}=0.8)$. The Wald CI suffers from violation of asymptotic properties of the normal approximation and delta method, which cannot provide accurate interval estimates even with several moderate sample size scenarios. According to the performances of three discussed CIs, with very small sample size (less than 40) and response rates close to 0 or 1, one should choose the exact CIs which can maintain CPs. With large enough sample size (more than 200), the Wald CI can be used to save computational cost. Otherwise, the bootstrap percentile CI should be considered.

# **RNT based on survival endpoints**

For RCTs with survival endpoints, RNT can be computed from the estimated survival probability at a specified time point $\tau$ of clinical interest. Let $\hat{S}_{E}\left( \tau\right)$ and $\hat{S}_{C}(\tau)$ denote the Kaplan-Meier estimated survival probabilities at time $\tau$ for the experimental and control groups, respectively. Then,

$${\hat{\mathrm{RNT}}}_{\mathrm{surv}}\left( \tau\right)=\frac{1}{\hat{S}_{C}(\tau)}-\frac{1}{\hat{S}_{E}(\tau)},$$

and the corresponding $100\left( 1-\alpha\right)\%$ CI has the form,

$${\hat{\mathrm{RNT}}}_{\mathrm{surv}}\left( \tau\right)\pm z_{1-\frac{\alpha}{2}}\sqrt{\frac{\mathrm{Var}\left( \hat{S}_{C}\left( \tau\right) \right)}{\hat{S}_{C}^{4}\left( \tau\right)}+\frac{\mathrm{Var}\left( \hat{S}_{E}\left( \tau\right) \right)}{\hat{S}_{E}^{4}\left( \tau\right)}},$$

where $\mathrm{Var}\left( \hat{S}_{E}\left( \tau\right) \right)$ and $\mathrm{Var}\left( \hat{S}_{C}\left( \tau\right) \right)$ are the corresponding variances.

To capture the entire profile of the treatment effect over time, the restricted mean survival time (RMST) can be used to evaluate the mean survival time truncated up to a specified time point $\tau$ [2-4]. The RMST up to time $\tau$ is equal to the area under the survival curve $S(t)$ from 0 to $\tau$,

$$\mathrm{RMST}\left( \tau\right)=E\left( \min\left( T, \tau\right) \right)= \int_{0}^{\tau} S\left( t \right)dt,$$

and its estimate $\hat{\mathrm{RMST}}(\tau)$ can be easily obtained by plugging in the Kaplan-Meier estimate $\hat{S}(t)$. The estimate of $\mathrm{RN}T_{\mathrm{RMST}}(\tau)$ is defined as

$${\hat{\mathrm{RNT}}}_{\mathrm{RMST}}\left( \tau\right)=\frac{1}{{\hat{\mathrm{RMST}}}_{C}\left( \tau\right)/\tau}-\frac{1}{{\hat{\mathrm{RMST}}}_{E}\left( \tau\right)/\tau} = \tau\left( \frac{1}{{\hat{\mathrm{RMST}}}_{C}(\tau)}-\frac{1}{{\hat{\mathrm{RMST}}}_{E}(\tau)} \right),$$

where ${\hat{\mathrm{RMST}}}_{E}\left( \tau\right)$ and ${\hat{\mathrm{RMST}}}_{C}\left( \tau\right)$ are the estimated RMSTs up to $\tau$ in the experimental and control arms, respectively. The $100\left( 1-\alpha\right)\%$ CI of ${\hat{\mathrm{RNT}}}_{\mathrm{RMST}}\left( \tau\right)$ can be constructed as

$${\hat{\mathrm{RNT}}}_{\mathrm{RMST}}\left( \tau\right)\pm z_{1-\frac{\alpha}{2}}\mathrm{SE}\left( {\hat{\mathrm{RNT}}}_{\mathrm{RMST}}\left( \tau\right) \right),$$

where

$$\mathrm{SE}({\hat{\mathrm{RNT}}}_{\mathrm{RMST}}\left( \tau) \right)=\tau\sqrt{\mathrm{Var}\left( \frac{1}{{\hat{\mathrm{RMST}}}_{C}\left( \tau\right)} \right)+\mathrm{Var}\left( \frac{1}{{\hat{\mathrm{RMST}}}_{E}\left( \tau\right)} \right)},$$

$$\mathrm{Var}\left( \frac{1}{{\hat{\mathrm{RMST}}}_{i}\left( \tau\right)} \right)=\frac{\mathrm{Var}\left( {\hat{\mathrm{RMST}}}_{i}\left( \tau\right) \right)}{\left( {\hat{\mathrm{RMST}}}_{i}\left( \tau\right) \right)^{4}}, i=E,C.$$

The $100(1-\alpha)\%$ CI of the $N\mathrm{RT}_{\mathrm{RMST}}\left( \tau\right)$ can be calculated by

$${\hat{\mathrm{RNT}}}_{\mathrm{RMST}}\left( \tau\right)\pm\tau z_{1-\frac{\alpha}{2}}\sqrt{\frac{\mathrm{Var}\left( {\hat{\mathrm{RMST}}}_{C}\left( \tau\right) \right)}{\left( {\hat{\mathrm{RMST}}}_{C}\left( \tau\right) \right)^{4}}+\frac{\mathrm{Var}\left( {\hat{\mathrm{RMST}}}_{E}\left( \tau\right) \right)}{\left( {\hat{\mathrm{RMST}}}_{E}\left( \tau\right) \right)^{4}}}.$$

With small sample size, the Wald CI may be narrow and fail to maintain the coverage probability. In such cases, we can approximate the bounds of CI for time-to-event data via a perturbation-resampling method [5]. For the observed time-to-event data $\left\{ X_{ij}=\min\left( T_{ij},C_{ij} \right),\Delta_{ij}=I(T_{ij}\leq C_{ij}),j=1,\ldots,n_{i} \right\}$, where $T_{ij}$ is the event time, $C_{ij}$ is the censoring time and $n_{i}$ is the number of observations of arm $i$ for $i=E,C$. The following steps can be used to estimate the CIs of RNT based on survival rates and RMSTs.

1. Given observations $\left\{ X_{Ej},\Delta_{Ej},j=1,\ldots,n_{E} \right\}$and $\left\{ X_{Cj},\Delta_{Cj},j=1,\ldots,n_{C} \right\},$for the $k$-th simulation, $k=1,\ldots, B,$ generate $\omega_{Ej}^{(k)}\sim\mathrm{Exp}\left( 1 \right),j=1,\ldots, n_{E}$; generate $\omega_{Cj}^{(k)}\sim\mathrm{Exp}\left( 1 \right),j=1,\ldots, n_{C}$.
2. Calculate $S_{E}^{\left( k \right)}\left( \tau\right), S_{C}^{\left( k \right)}\left( \tau\right), \mathrm{RMST}_{E}^{\left( k \right)}\left( \tau\right)$ and$\mathrm{RMST}_{C}^{\left( k \right)}\left( \tau\right)$ for the prespecified time point $\tau$ based on weighted time-to-event data which uses ${\{\omega}_{Ej}^{(k)},j=1,\ldots, n_{E}\}$ and ${\{\omega}_{Cj}^{(k)},j=1,\ldots, n_{C}\}$as weights.
3. Calculate $\mathrm{RN}T_{\mathrm{surv}}^{(k)}\left( \tau\right)$ and $\mathrm{RN}T_{\mathrm{RMST}}^{(k)}\left( \tau\right)$.
4. The estimated lower and upper bounds of ${\hat{\mathrm{RNT}}}_{\mathrm{surv}}\left( \tau\right)$ and ${\hat{\mathrm{RNT}}}_{\mathrm{RMST}}\left( \tau\right)$ are approximated by the $\left( \frac{\alpha}{2} \right)$-th and $\left( 1-\frac{\alpha}{2} \right)$-th empirical quantiles of $\left\{ \mathrm{RN}T_{\mathrm{surv}}^{(k)}\left( \tau\right),k=1,\ldots,B \right\}$ and $\left\{ \mathrm{RN}T_{\mathrm{RMST}}^{(k)}\left( \tau\right),k=1,\ldots,B \right\}$, respectively.

We conducted simulations to examine the performances of the Wald and perturbation-resampling CIs based on survival rates and RMSTs with results shown in Supplementary Table S2. With regard to the event time distributions for the experimental and control arms, we considered Weibull distributions with different shape and scale parameters. For each scenario, we set the study ending time to be 1 and assumed the censoring distribution be an exponential distribution for which the rate parameter was chosen to yield a 15% non-administrative censoring rate. The RNTs based on survival rates and RMSTs were calculated at $\tau=1.$

**Table S2. Survival endpoint simulation results for coverage probabilities of the Wald, perturbation-resampling confidence intervals of** $\mathbf{RN}\mathbf{T}_{\mathbf{surv}}$ **and** $\mathbf{RN}\mathbf{T}_{\mathbf{RMST}}$**.**

|  | Weibull($\lambda,k)$ | | $\mathrm{RN}T_{\mathrm{surv}}$ | | $\mathrm{RN}T_{\mathrm{RMST}}$ | |
| --- | --- | --- | --- | --- | --- | --- |
| $n$ | Experimental | Control | CP (Wald) | CP (Resampling) | CP (Wald) | CP (Resampling) |
| 20 | (0.8,0.5) | (1,0.5) | 0.999 | 0.934 | 0.985 | 0.932 |
| 20 | (1.5,0.5) | (1,0.5) | 0.998 | 0.940 | 0.985 | 0.934 |
| 20 | (1,1) | (1,0.5) | 1.000 | 0.935 | 0.966 | 0.933 |
| 20 | (0.8,1) | (1,1) | 0.994 | 0.933 | 0.964 | 0.933 |
| 20 | (1,1) | (1,1) | 1.000 | 0.935 | 0.971 | 0.931 |
| 20 | (1.5,1) | (1,1) | 0.981 | 0.943 | 0.966 | 0.938 |
| 20 | (0.8,2) | (1,2) | 0.956 | 0.928 | 0.952 | 0.932 |
| 20 | (1,1) | (1,2) | 1.000 | 0.935 | 0.948 | 0.933 |
| 20 | (1.5,2) | (1,2) | 0.935 | 0.947 | 0.951 | 0.942 |
| 100 | (0.8,0.5) | (1,0.5) | 0.971 | 0.946 | 0.958 | 0.946 |
| 100 | (1,1) | (1,0.5) | 0.973 | 0.944 | 0.954 | 0.948 |
| 100 | (1.5,0.5) | (1,0.5) | 0.970 | 0.950 | 0.959 | 0.946 |
| 100 | (0.8,1) | (1,1) | 0.972 | 0.947 | 0.952 | 0.945 |
| 100 | (1,1) | (1,1) | 0.971 | 0.946 | 0.956 | 0.947 |
| 100 | (1.5,1) | (1,1) | 0.959 | 0.947 | 0.951 | 0.943 |
| 100 | (0.8,2) | (1,2) | 0.956 | 0.951 | 0.951 | 0.945 |
| 100 | (1,1) | (1,2) | 0.971 | 0.945 | 0.952 | 0.944 |
| 100 | (1.5,2) | (1,2) | 0.945 | 0.950 | 0.947 | 0.949 |
| 500 | (0.8,0.5) | (1,0.5) | 0.953 | 0.949 | 0.950 | 0.947 |
| 500 | (1,1) | (1,0.5) | 0.953 | 0.946 | 0.949 | 0.946 |
| 500 | (1.5,0.5) | (1,0.5) | 0.952 | 0.946 | 0.947 | 0.944 |
| 500 | (0.8,1) | (1,1) | 0.957 | 0.951 | 0.949 | 0.946 |
| 500 | (1,1) | (1,1) | 0.955 | 0.948 | 0.948 | 0.947 |
| 500 | (1.5,1) | (1,1) | 0.952 | 0.946 | 0.947 | 0.944 |
| 500 | (0.8,2) | (1,2) | 0.951 | 0.949 | 0.948 | 0.945 |
| 500 | (1,1) | (1,2) | 0.955 | 0.950 | 0.948 | 0.946 |
| 500 | (1.5,2) | (1,2) | 0.954 | 0.950 | 0.951 | 0.949 |

CP represents the coverage probability; $n$ is the sample size; Weibull($\lambda,k)$ represents the Weibull distribution with scale $\lambda$ and shape $k$; RNTs based on survival rates and RMSTs were evaluated at $\tau=1$.

The perturbation-resampling CIs of $\mathrm{RN}T_{\mathrm{surv}}$ and $\mathrm{RN}T_{\mathrm{RMST}}$ yield accurate CPs close to the nominal level 95% regardless of sample sizes and event time distributions. The Wald CIs might be too conservative (CPs larger than 0.97) with small sample size, especially for $\mathrm{RN}T_{\mathrm{surv}}$. The standard errors of Kaplan-Meier survival rates are usually computed on the log or complementary log-log scale and then obtained by the delta method. The asymptotic normality of $\mathrm{RN}T_{\mathrm{surv}}$ after such complex transformation may not hold without adequate sample size.

# **Detailed estimates and 95% CIs of** $\mathbf{RNT}$ **and** $\mathbf{NNT}$ **for Examples 2 to 4**

**Table S3. Estimates and 95% CIs of** $\mathbf{RN}\mathbf{T}_{\mathbf{surv}}$ **and** $\mathbf{NN}\mathbf{T}_{\mathbf{surv}}$ **for the S0226 trial [6].**

| Time (years) | $\mathrm{RN}T_{\mathrm{surv}}$ (Wald) | $\mathrm{RN}T_{\mathrm{surv}}$ (Resampling) | $\mathrm{NN}T_{\mathrm{surv}}$ |
| --- | --- | --- | --- |
| 1 | 0.03 [-0.21,0.26] | 0.03 [-0.22,0.27] | 125.82 [12.23 to $\infty$ to 15.17] |
| 2 | 0.74 [-0.03,1.50] | 0.74 [-0.03,1.58] | 14.60 [7.30 to 10526] |
| 3 | 2.52 [0.69,4.35] | 2.52 [0.89,4.60] | 10.84 [6.62 to 29.85] |
| 4 | 5.89 [1.69,10.09] | 5.89 [2.32,11.42] | 11.22 [7.19 to 25.54] |
| 5 | 9.04 [1.99,16.08] | 9.04 [3.36,18.67] | 12.94 [8.19 to 30.83] |
| 6 | 7.28 [-0.03,14.59] | 7.28 [0.92,16.94] | 20.00 [10.79 to 136.2] |
| 7 | 9.04 [-1.28,19.37] | 9.04 [0.22,23.49] | 24.18 [12.27 to 829.2] |
| 8 | 7.27 [-5.75,20.29] | 7.27 [-5.02,25.74] | 44.48 [16.56 to $\infty$ to 64.89] |
| 9 | 9.46 [-7.67,26.60] | 9.46 [-6.77,36.36] | 43.86 [16.57 to $\infty$ to 67.87] |
| 10 | 35.11 [-42.67,112.9] | 35.11 [1.06,996.22] | 23.64 [11.92 to 1497] |

**Table S4. Estimates and 95% CIs of** $\mathbf{RNT}_{\mathbf{surv}}$ **and** $\mathbf{NNT}_{\mathbf{surv}}$ **for the urgent versus early endoscopy trial [7].**

| Time (days) | $\mathrm{RN}T_{\mathrm{surv}}$ (Wald) | $\mathrm{RN}T_{\mathrm{surv}}$ (Resampling) | $\mathrm{NN}T_{\mathrm{surv}}$ |
| --- | --- | --- | --- |
| 5 | 0.012 [-0.021,0.045] | 0.012 [-0.022,0.047] | 86.00 [23.58 to $\infty$ to 52.20] |
| 10 | 0.000 [-0.042,0.042] | 0.000 [-0.043,0.043] | $\infty$ [26.49 to $\infty$ to 26.49] |
| 15 | -0.017 [-0.063,0.028] | -0.017 [-0.066,0.028] | -64.50 [40.23 to $\infty$ to 17.90] |
| 20 | -0.022 [-0.072,0.028] | -0.022 [-0.074,0.028] | -51.60 [41.70 to $\infty$ to 15.94] |
| 25 | -0.027 [-0.08,0.026] | -0.027 [-0.083,0.026] | -43.00 [45.93 to $\infty$ to 14.64] |
| 30 | -0.027 [-0.082,0.027] | -0.027 [-0.085,0.027] | -43.00 [43.77 to $\infty$ to 14.42] |

**Table S5. Estimates and 95% CIs of** $\mathbf{RNT}_{\mathbf{RMST}}$ **and** $\mathbf{NNT}_{\mathbf{RMST}}$ **for the urgent versus early endoscopy trial [7].**

| Time (days) | $\mathrm{RN}T_{\mathrm{RMST}}$ (Wald) | $\mathrm{RN}T_{\mathrm{RMST}}$ (Resampling) | $\mathrm{NN}T_{\mathrm{RMST}}$ |
| --- | --- | --- | --- |
| 5 | 0.006 [-0.015,0.028] | 0.006 [-0.015,0.029] | 172.0 [37.79 to $\infty$ to 67.41] |
| 10 | 0.008 [0.019,0.036] | 0.008 [-0.018,0.037] | 125.9 [29.85 to $\infty$ to 56.77] |
| 15 | 0.004 [-0.027,0.035] | 0.004 [-0.028,0.036] | 286.7 [30.95 to $\infty$ to 39.47] |
| 20 | -0.002 [-0.036,0.032] | -0.002 [-0.035,0.035] | -543.2 [35.75 to $\infty$ to -26.28] |
| 25 | -0.006 [-0.042,0.031] | -0.006 [-0.042,0.031] | -198.5 [33.50 to $\infty$ to 25.49] |
| 30 | -0.009 [-0.047,0.030] | -0.009 [-0.048,0.031] | -127.9 [37.49 to $\infty$ to 23.64] |

**Table S6. Estimates and 95% CIs of** $\mathbf{RN}\mathbf{T}_{\mathbf{surv}}$ **and** $\mathbf{NN}\mathbf{T}_{\mathbf{surv}}$ **for the prophylactic cranial irradiation trial [8].**

| Time (years) | $\mathrm{RN}T_{\mathrm{surv}}$ (Wald) | $\mathrm{RN}T_{\mathrm{surv}}$ (Resampling) | $\mathrm{NN}T_{\mathrm{surv}}$ |
| --- | --- | --- | --- |
| 1 | 0.13 [-0.22,0.49] | 0.13 [-0.24,0.50] | 25.20 [6.84 to $\infty$ to 14.98] |
| 2 | 0.41 [-0.49,1.31] | 0.41 [-0.50,1.37] | 21.69 [6.79 to $\infty$ to 18.15] |
| 3 | 1.35 [-0.27,2.96] | 1.35 [-0.14,3.29] | 12.16 [5.71 to $\infty$ to 93.90] |
| 4 | 1.20 [-0.98,3.38] | 1.20 [-0.96,3.82] | 20.42 [7.37 to $\infty$ to 26.44] |
| 5 | 0.97 [-1.76,3.69] | 0.97 [-1.89,4.15] | 33.92 [8.96 to $\infty$ to 18.97] |
| 6 | 0.90 [-2.25,4.05] | 0.90 [-2.48,4.51] | 43.81 [9.79 to $\infty$to 17.71] |
| 7 | 0.74 [-2.72,4.20] | 0.74 [-3.02,4.61] | 60.25 [10.64 to $\infty$ to 16.44] |
| 8 | 3.01 [-2.21,8.23] | 3.01 [-1.91,9.74] | 22.50 [8.59 to $\infty$ to 36.37] |
| 9 | 3.90 [-3.12,10.92] | 3.90 [-2.37,13.77] | 24.13 [9.13 to $\infty$ to 37.48] |
| 10 | 5.38 [-2.92,13.69] | 5.38 [-1.59,18.08] | 19.67 [8.47 to $\infty$ to 60.67] |

**Table S7. Estimates and 95% CIs of** $\mathbf{RN}\mathbf{T}_{\mathbf{RMST}}$ **and** $\mathbf{NN}\mathbf{T}_{\mathbf{RMST}}$ **for the prophylactic cranial irradiation trial [8].**

| Time (years) | $\mathrm{RN}T_{\mathrm{RMST}}$ (Wald) | $\mathrm{RN}T_{\mathrm{RMST}}$ (Resampling) | $\mathrm{NN}T_{\mathrm{RMST}}$ |
| --- | --- | --- | --- |
| 1 | 0.03 [-0.09,0.15] | 0.03 [-0.09,0.16] | 52.56 [11.48 to $\infty$ to 20.40] |
| 2 | 0.12 [-0.11,0.34] | 0.12 [-0.11,0.34] | 25.25 [8.60 to $\infty$ to 26.98] |
| 3 | 0.23 [-0.10,0.56] | 0.23 [-0.11,0.56] | 18.68 [7.63 to $\infty$ to 41.56] |
| 4 | 0.31 [-0.12,0.75] | 0.31 [-0.11,0.76] | 18.09 [7.60 to $\infty$ to 47.65] |
| 5 | 0.38 [-0.16,0.91] | 0.38 [-0.16,0.93] | 19.08 [7.84 to $\infty$ to 44.11] |
| 6 | 0.42 [-0.23,1.06] | 0.42 [-0.24,1.07] | 20.87 [8.23 to $\infty$ to 38.98] |
| 7 | 0.44 [-0.31,1.19] | 0.44 [-0.30,1.22] | 23.19 [8.66 to $\infty$ to 34.13] |
| 8 | 0.49 [-0.35,1.34] | 0.49 [-0.35,1.36] | 24.02 [8.87 to $\infty$ to 34.00] |
| 9 | 0.58 [-0.37,1.53] | 0.58 [-0.41,1.56] | 23.66 [8.97 to $\infty$ to 37.13] |
| 10 | 0.67 [-0.39,1.73] | 0.67 [-0.35,1.76] | 23.21 [9.05 to $\infty$ to 41.15] |


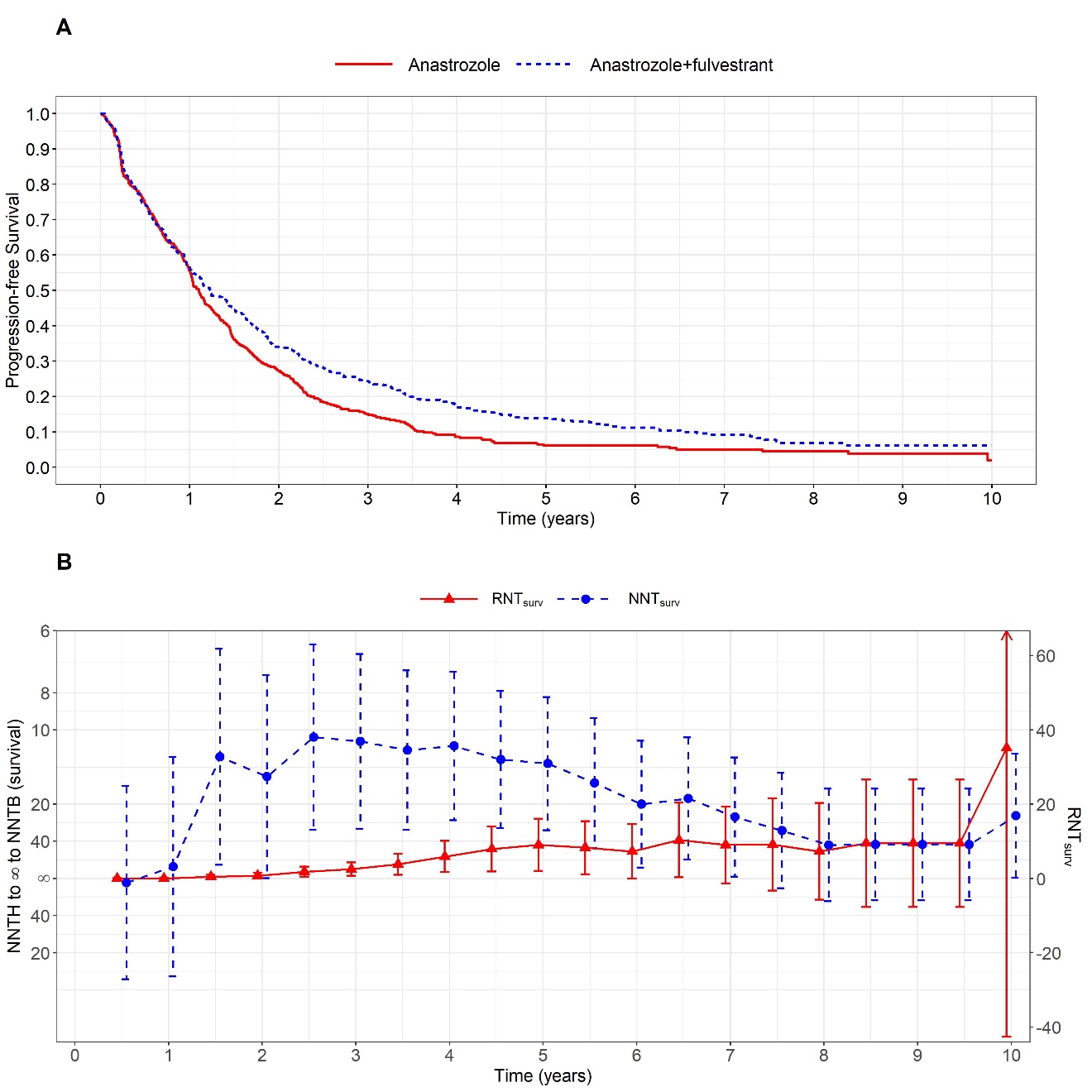


**Figure S1.** **NNT vs RNT (Wald CIs) for the S0226 trial [6].** (A) Kaplan-Meier estimates of progression-free survival curves for the fulvestrant plus anastrozole therapy and anastrozole therapy alone; (B) NNTs and RNTs calculated from survival rates with their 95% CIs.


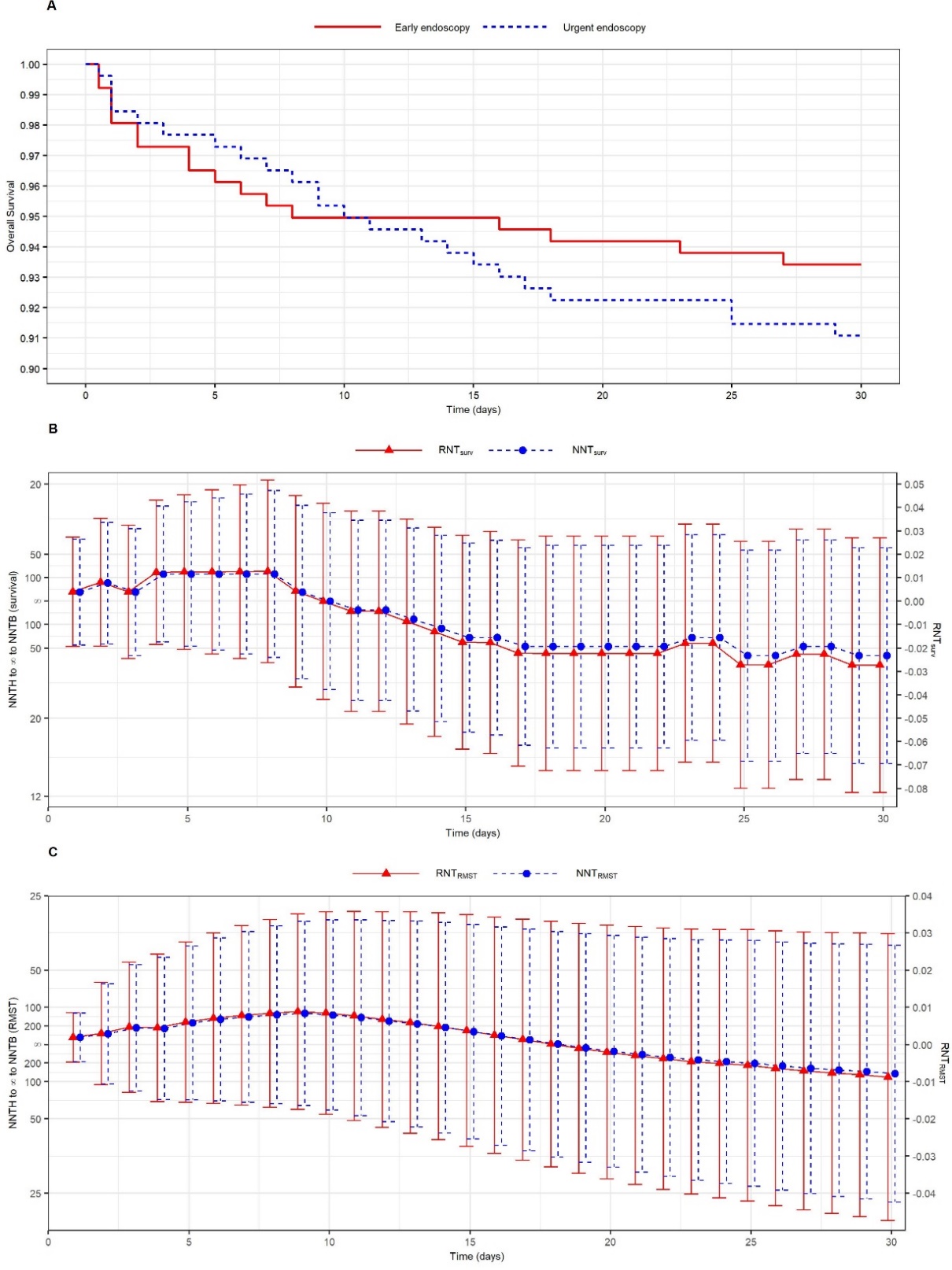


**Figure S2. NNT vs RNT (Wald CIs) from the urgent versus early endoscopy trial [7].** (A) Kaplan-Meier estimates of overall survival curves for the urgent endoscopy and early endoscopy groups; (B) NNTs and RNTs calculated from the survival rates with their 95% CIs; (C) NNTs and RNTs calculated from RMST with their 95% CIs.


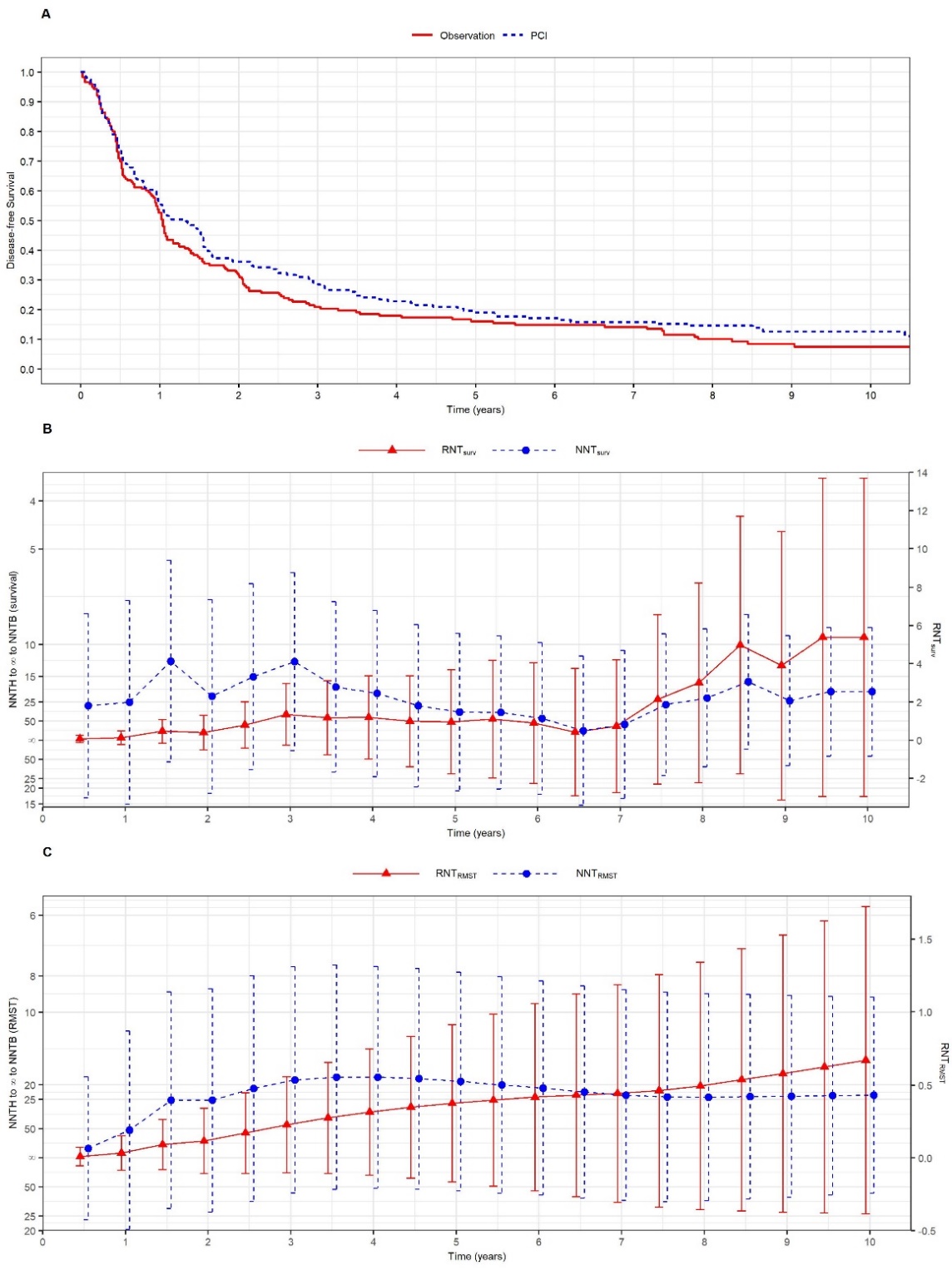


**Figure S3. NNT vs RNT (Wald CIs) for the prophylactic cranial irradiation trial [8].** (A) Kaplan-Meier estimates of disease-free survival curves for the prophylactic cranial irradiation (PCI) and observation groups; (B) NNTs and RNTs calculated from the survival rates with their 95% CIs; (C) NNTs and RNTs calculated from RMST with their 95% CIs.

**Supplementary References**

1. Agresti A: **Categorical data analysis**, vol. 482: John Wiley & Sons; 2003.

2. Uno H, Claggett B, Tian L, Inoue E, Gallo P, Miyata T, Schrag D, Takeuchi M, Uyama Y, Zhao L *et al*: **Moving beyond the hazard ratio in quantifying the between-group difference in survival analysis**. *J Clin Oncol* 2014, **32**(22):2380-2385.

3. Uno H, Wittes J, Fu H, Solomon SD, Claggett B, Tian L, Cai T, Pfeffer MA, Evans SR, Wei LJ: **Alternatives to Hazard Ratios for Comparing the Efficacy or Safety of Therapies in Noninferiority Studies**. *Ann Intern Med* 2015, **163**(2):127-134.

4. Royston P, Parmar MK: **Restricted mean survival time: an alternative to the hazard ratio for the design and analysis of randomized trials with a time-to-event outcome**. *BMC Med Res Methodol* 2013, **13**:152.

5. Zhao L, Claggett B, Tian L, Uno H, Pfeffer MA, Solomon SD, Trippa L, Wei LJ: **On the restricted mean survival time curve in survival analysis**. *Biometrics* 2016, **72**(1):215-221.

6. Mehta RS, Barlow WE, Albain KS, Vandenberg TA, Dakhil SR, Tirumali NR, Lew DL, Hayes DF, Gralow JR, Linden HM *et al*: **Overall Survival with Fulvestrant plus Anastrozole in Metastatic Breast Cancer**. *New England Journal of Medicine* 2019, **380**(13):1226-1234.

7. Lau JYW, Yu Y, Tang RSY, Chan HCH, Yip HC, Chan SM, Luk SWY, Wong SH, Lau LHS, Lui RN *et al*: **Timing of Endoscopy for Acute Upper Gastrointestinal Bleeding**. *N Engl J Med* 2020, **382**(14):1299-1308.

8. Sun A, Hu C, Wong SJ, Gore E, Videtic G, Dutta S, Suntharalingam M, Chen Y, Gaspar LE, Choy H: **Prophylactic Cranial Irradiation vs Observation in Patients With Locally Advanced Non-Small Cell Lung Cancer: A Long-term Update of the NRG Oncology/RTOG 0214 Phase 3 Randomized Clinical Trial**. *JAMA Oncol* 2019, **5**(6):847-855.
